# The Current State of Donor-Derived Cell-Free DNA Use in Allograft Monitoring in Kidney Transplantation

**DOI:** 10.3390/jpm12101700

**Published:** 2022-10-12

**Authors:** Michael L. Kueht, Laxmi Priya Dongur, Matthew Cusick, Heather L. Stevenson, Muhammad Mujtaba

**Affiliations:** 1 Department of Surgery, Multiorgan Transplant and Hepatobiliary Surgery, University of Texas Medical Branch, Galveston, TX 77555, USA; 2 Department of Pathology, Division of Histocompatibility and Immunogenetics, University of Michigan Medicine, Ann Arbor, MI 48109, USA; 3 Department of Pathology, Transplant Pathology, University of Texas Medical Branch, Galveston, TX 77555, USA; 4 Department of Medicine, Transplant Nephrology, University of Texas Medical Branch, Galveston, TX 77555, USA

**Keywords:** renal transplantation, T-cell mediated rejection, antibody-mediated rejection, do-nor-derived cell-free DNA

## Abstract

Renal transplantation is the definitive therapy for patients suffering from end-stage renal disease. Though there have been significant advances in immunosuppression in these patients, there is still up to 30% acute and subclinical rejection. Current standards employ lab markers of renal function and biopsy results for accurate diagnosis. However, donor derived cell-free DNA has been identified as a measurable lab test that may be able to adequately diagnose rejection at early stages, precluding the need for invasive procedures like biopsy. We obtained published data directly from companies that offer ddcfDNA assay tests and additionally conducted a literature review using databases like PUBMED and NIH U.S. National Library of Medicine. We comprehensively compare the most used ddcfDNA assays, delineate their respective limitations, and further explore future directions in the utility of ddcfDNA in renal transplant patients.

## 1. Introduction

**Figure 1:**
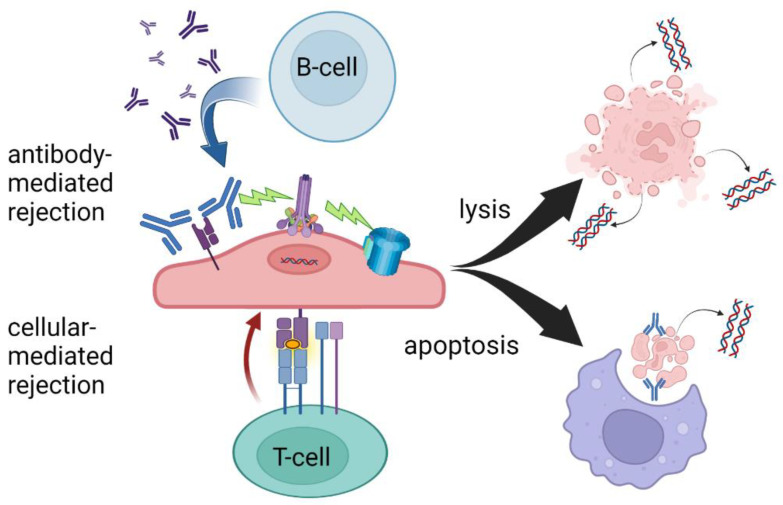
T-cell- and antibody-mediated rejection leading to cell lysis and/or apoptosis. While there is significant overlap in the mechanisms of AMR and TCMR, ddcfDNA levels may be more sensitive to AMR due to increased circulating cellular debris following cell lysis. Created with BioRender.com.

Clinical kidney transplantation has evolved from a niche operation that was historically only possible between identical twins at a few highly specialized centers to a standard of care for patients with end-stage renal disease that can be conducted between donor–recipient pairs from disparate ethnicities and geographies. As the complexities of donor–recipient matching increase alongside our understanding of the highly variable human immune system, more complex methods of allograft monitoring are needed beyond standard measures of serum creatine and proteinuria. Indeed, despite the routine use of induction and multi-drug maintenance immunosuppression, expected incidences of acute and subclinical rejection can be up to 20–30% and these data come from biopsy-proven pathology [1]. The quest for specific and reproducible noninvasive markers of graft injury has naturally led to genetic markers including donor-derived cell-free DNA (ddcfDNA), released during injury and rejection of an allograft (Figure 1). Here, we describe some of the historical advances leading to ddcfDNA as a clinical tool to aid in the diagnosis of rejection in kidney transplantation, review the current uses and limitations, and explore some of the future directions of this exciting biomarker.

### 1.1. History of ddcfDNA in Kidney Transplantation

Although the permanent admixture of donor cellular material into the recipient system in organ transplantation was indirectly suspected as early as 1963 as playing a role in rejection and tolerance, the first direct evidence of DNA micro-chimerism in solid organ transplantation came in 1993 by Starzl et al. [2,3]. This was made feasible by developing methods to measure the presence of the genetic material of chromosomes 6 (Human Leukocyte Antigen, HLA) and Y (male sex), detecting mismatched donor-derived cells distant from the transplanted organ. The transition from cell-based DNA interrogation (probe-based cytostaining and cellular homogenates) to cell-free DNA detection was made in 1998 by Lo and colleagues when they were able to isolate Y chromosome genetic material from the circulating plasma of female transplant recipients [4]. Despite the limited applicability of the approach (only useful in female recipients of organs from male donors), this represented the first donor-specific cell-free DNA (ddcfDNA) detection in solid organ transplantation and an early demonstration that reliable assumptions about donor genotype could be useful. Further advances in genomic sequencing led to sex-independent methods of distinguishing between the donor and recipient DNA that initially focused on HLA-specific quantitative PCR. Although this approach characterized a significant advance in amplifying very low levels of circulating ddcfDNA, the reproducibility was suboptimal, and the technique suffered from the limitation of not being able to distinguish well between HLA-matched recipient–donor pairs [5]. In 2011, Snyder et al. from Stanford University published a very reliable method for detecting ddcfDNA wherein microfluidic digital PCR was utilized to detect differences in single nucleotide polymorphism (SNP) frequencies between donor and recipient. This method was extremely accurate; however, required full genome sequencing of both the donor and the recipient, a resource and time-intensive endeavor [6]. Nonetheless, in this study, ddcfDNA was significantly correlated with acute rejection in heart transplant recipients and developed the backbone for all commercially available ddcfDNA tests today.

The next major advance toward ddcfDNA becoming a clinically useful tool in organ transplantation came from advances in computational genetics and the developing understating of population-level allelic frequencies stemming from the Human Genome Project [7,8]. In 2016, Sharon et al. showed that useful measures of ddcfDNA could be obtained in the absence of full genotyping of the donor by analyzing 150–600 thousand SNPs. This method utilized full genotyping of the recipient and was able to distinguish between related and unrelated donor–recipient pairs utilizing the principles of allelic equilibrium and chromosomal inheritance [9]. That same year, Grskovic et al. showed the feasibility of quantifying ddcfDNA with neither a full recipient nor a full donor genotype by interrogating just 266 SNPs [10]. These polymorphisms were targeted due to an extremely low probability of two unrelated individuals having identical genotypes and a low linkage state. Although this method performed equally well in closely and distantly related pairings, it was limited in its ability to identify the presence of more than two distinct genomes as might be found in recipients of transplants from multiple donors. Furthermore, as in all DNA testing, there remained an inability to detect ddcfDNA from monozygotic (identical) twins.

From these myriad advancements, we are able today to utilize ddcfDNA as a practical clinical assay, available via several commercial preparations and approved by many insurance plans to aid in the diagnosis of allograft rejection. Nonetheless, limitations persist in distinguishing among etiologies of allograft injury and our understanding of the optimal use of these powerful tests is constantly evolving.

### 1.2. Overview of Commercially Available ddcfDNA Tests

Today in the United States, three commercially available ddcfDNA assays are available for clinical use in kidney transplantation, *Allosure* by CareDx, *Prospera* by Natera, and *TRAC* by Viracor Eurofins [11,12,13]. These tests all currently require whole blood to be analyzed in a centralized laboratory, a “send out” test, and differ mainly in the number of SNPs measured. At the time of development of these assays, the gold standard for diagnosing rejection was the interpretation of histopathology by a trained pathologist according to the Banff criteria and therefore, all tests were initially validated based on biopsy-proven rejection via classic histology findings [14]. The ubiquitous finding, regardless of platform, that elevated ddcfDNA better predicts antibody-mediated rejection (AMR) compared to T-cell-mediated rejection is a yet poorly understood phenomenon, see Table 1. At the validated thresholds, ddcfDNA is more sensitive to antibody-mediated rejection. This may be due to complement-activated recruitment of the membrane attack complex leading to cell lysis and hence the release of more intracellular debris including cfDNA. The targeting of the microvasculature endothelium may also create an ischemic environment contributing to necrosis. In the setting of T-cell-mediated rejection, phagocytosis following apoptosis may sequester more intracellular contents, leading to less measurable cfDNA despite the presence of graft injury (Figure 1).

## 2. Current Uses

The tests have evolved to be employed in conjunction with tests such as circulating leukocyte gene expression markers due in part to the fact that they all share relatively high negative predictive values (NPV) and relatively low positive predictive values (PPV). In short, the absence of ddcfDNA is a better predictor of the state of an allograft, in terms of rejection, than the presence of ddcfDNA, which may represent driving forces other than rejection. Outside of the landmark original validation studies, this finding has been reproduced by several groups, with NPV being consistently higher than PPV [18,19,20,21,22]. Additionally, the clinical performance of commercially available ddcfDNA tests appears to be similar (commonly used cutoff value of ~1%) despite different validation strategies [23,24]. Therefore, the main utility in the clinical setting, consistent with the original intention, is to confidently rule out suspected rejection and avoid a potentially unnecessary biopsy. Indeed, with the rare but potentially catastrophic complications of percutaneous biopsy combined with the inherent diagnostic limitations, avoiding even one biopsy in the lifetime of a kidney recipient represents a substantial improvement [25].

### Limitations to Current Use

The relatively low PPV of using ddcfDNA alone to confirm the diagnosis of acute rejection makes an elevated ddcfDNA level difficult to interpret in the absence of additional information. As nonrejection pathology may elevate ddcfDNA levels, it is important to have a comprehensive clinical picture of the patient for decision-making [26]. In a retrospective study from 2020, Goussous et al. illustrate instances of elevations in ddcfDNA with concomitant BK viremia; however, the incidence was not high enough for statistical significance [27]. Kant et al. further investigated the effect of BK viremia and simultaneous rejection on the levels of ddcfDNA. Though a positive correlation was demonstrated between BK viremia and ddcfDNA levels from a small cohort of 10 patients, there was no significant effect of rejection in patients with BK viremia on ddcfDNA levels [28]. While cellular injury is deemed to be the perpetrator behind elevation in ddcfDNA, there is currently not enough research to delineate the pathophysiology behind nonrejection mediated insults that affect ddcfDNA levels.

Additionally, as initial validation studies were performed on single timepoints wherein rejection was suspected and a biopsy was performed, appropriate ddcfDNA levels for given periods of time post-transplant are not well understood. Indeed, many recipient factors (e.g., panel reactive antibody [PRA], repeat transplant status) interact with donor factors (e.g., donation after circulatory death [DCD]) over time to influence ddcfDNA levels in the absence of suspicion of rejection [29]. Sureshkumar et al. in 2020 conducted a retrospective study that evaluated the influence of obesity on ddcfDNA levels. While morbid obesity is noted to have an inverse relationship with ddcfDNA levels ( R = 0.29, R^2^ = 0.089, *p* = 0.001), the interaction with increased adipose tissue and ddcfDNA is not well understood [30]. Other scenarios in which the value of ddcfDNA remains unknown include long-term (>10 yr) stable graft function and values outside several standard deviations from expected. Attempts to establish a range of ‘normal’ ddcfDNA in transplant recipients without suspicion for rejection reported a median value of 0.23% (interquartile range 0.12–0.39%) with a large range, up to 1.2% [31]. As many of these ‘normal’ values are above suggested cutoffs to aid in the diagnosis of rejection, the value of ddcfDNA in a recipient with good and stable graft function remains unclear.

## 3. Future Directions

As experience with ddcfDNA expands, concerted efforts to refine the optimal use of this test are increasing and include exploiting the NPV by serially measuring levels to monitor the response to treatment for chronic rejection, facilitate the transition of immunosuppression regimens, and identify states of graft immuno-quiescence (Table 2).

### 3.1. Monitoring Response to Treatment for Chronic Rejection

The use of serial measurements of ddcfDNA in monitoring the response to treatment for rejection has been previously suggested as a feasible adjunct to post-treatment biopsy [32]. Researchers from Cedars-Sinai Medical Center are currently evaluating the utility of ddcfDNA (NCT03859388) in assessing treatment response to chronic antibody-mediated rejection (ABMR). Proper diagnosis of ABMR involves anti-HLA donor-specific antibodies (DSA) in addition to a biopsy with histologic confirmation. Based on current standards, DSA, serum creatinine, and renal biopsies are used to evaluate for treatment response. However, only a small proportion of patients with ABMR will have a decrease in DSA in response to treatment. Furthermore, patients with chronic ABMR can have high levels of DSA despite adequate renal function and reversal of histological evidence of rejection on biopsy. By following longitudinal changes in ddcfDNA after monthly administration of tocilizumab, the group hopes to expand the use of ddcfDNA to not only rejection surveillance but also treatment response surveillance. The study is currently finished with the recruitment phase. Results are expected in the upcoming year.

### 3.2. Facilitating Transition of Immunosuppression Regimens

The transition from calcineurin inhibitor-based maintenance immunosuppression to avoid long-term nephrotoxicity has been an enticing goal since the results of the BENEFIT trial showed superior GFRs attained at 3-7 years post-transplant [33]. A group from the University of Texas Southwestern Medical Center hopes to evaluate the efficacy of ddcfDNA in monitoring Belatacept therapy response, whilst concurrently assessing the effectiveness of Belatacept alone versus multi-drug immunosuppression (NCT04786067). Participants will undergo surveillance with ddcfDNA measurements and peripheral leukocyte gene expression testing. The primary outcome of acute kidney graft rejection will be confirmed by protocol biopsy at 12 months. The study is currently recruiting and is expected to publish preliminary data in 2023.

### 3.3. Identifying Graft Immuno-Quiescence

As an alternative to identify suspected rejection, a group of investigators sponsored by CareDx hopes to use ddcfDNA to identify an immune-quiescent state in stable individuals. The ADMIRAL study (NCT04566055), a multicenter observational study, aims to validate established clinical trial data to identify the effectiveness of ddcfDNA in early detection of rejection as well as a predictor of long-term graft survival. Preliminary data demonstrated that although there was no statistical significance between serum creatinine between no rejection and biopsy-proven rejection groups (median serum creatinine 1.38 mg/dL and 1.57 mg/dL, respectively, *p* = 0.1), there were significantly lower levels of ddcfDNA in the no rejection cohort compared to the biopsy-proven rejection group. Additionally, lowering the threshold to 0.5%, ddcfDNA was associated with subsequent development of de novo DSA (*p* < 0.01). The preliminary data are encouraging regarding the utility of ddcfDNA in surveillance and detection of rejection in early subclinical stages.

## 4. Summary

Given that immunosuppression to some extent is needed outside of identical twin transplants, balancing the desired effects and side/adverse effects of these narrow therapeutic drugs is a large part of the art and science of transplantation. The introduction of ddcfDNA into the toolkit of the transplant professional is a great addition to the science behind managing transplant recipients, although the use of this tool requires a certain degree of art in its current form. Like so many advances in the field rooted in biology and logic, including early methods of immunosuppression, the real-world experiences of practitioners are necessary to identify optimally applicable scenarios. As discovery sheds more and more light on the complexities of human immunology, we are frequently faced with the fact that “one size does not fit all”. In the spirit of helping as many people as possible, it is necessary to continue to use evolving standards of care while at the same time pushing the boundaries of what is possible, and so it is with ddcfDNA. The power to exploit the properties of DNA including its specificity and traceability through ancestral lineages makes ddcfDNA a very promising tool in transplantation and it is no surprise that we have yet to find the optimal implementation. Additionally, it is important to balance the costs, both monetary and otherwise, of testing with the principles of beneficence and non-maleficence when it comes to unintended consequences to patients.

The future of ddcfDNA in kidney transplantation is likely an integral part of multi-faceted testing strategies that encompass DSA (both anti-HLA and non-HLA), gene expression assays (tissue and peripheral blood), advanced urinary analyses, and yet undiscovered methodologies. As we continue to strive to provide the best care possible for transplant recipients, it is incumbent on the transplant field to utilize available technologies while simultaneously questioning dogma and possessing a willingness to change practices as evidence develops.

## Figures and Tables

**Table 1:** Summary of characteristics of commercially available clinical ddcfDNA tests in the US for use in transplant recipients with suspicion of acute rejection.

	AlloSure (CareDx)	Prospera (Natera)	TRAC (Viracor Eurofins)
Initial Validation Cohort	DART (NCT01299168, 2015–2016) [15] prospective 14 sites 102 patients	UCSF biobank (pre-2018) * [16] retrospective 1 site 178 patients	Undisclosed biobank (pre-2020) ** [17] retrospective 1 site 25 patients
Calibration Standard	Histopathology (BPR)	Histopathology (BPR)	Histopathology (BPR)
Targeted diagnosis	Acute rejection (AMR > TCR)	Acute rejection (AMR > TCR)	Acute rejection (AMR > TCR)
Suggested threshold	1%	1%	0.7%
Reported sensitivity, specificity	59%, 85%	89%, 73%	58%, 85%
NPV, PPV ***	84%, 61%	95%, 52%	86%, 55%
Potential false positive rate ***	15%	27%	15%

DNA in Blood for Diagnosing Active Rejection in Kidney Transplant Recipients (DART) Study; UCSF, University of California San Francisco; BPR, biopsy-proven rejection; AMR, antibody-mediated rejection; TCR, T-cell-mediated rejection; NPV, negative predictive value; PPV, positive predictive value. * Study duration not included in landmark publication, subsequently studied in TRIFECTA (NCT04239703, 2019-recruiting, *n* = 367). ** initially studied in R&D setting, subsequently studied post hoc via CTOT-08 (NCT01289717, 2011-2016) and single center biobank (Northwestern University). *** at 25% prevalence.

**Table 2:** Current clinical trials investigating ddcfDNA.

Study Name and Design	Institution	Cohort	Exclusion Criteria	Primary Outcome	Secondary Outcome	Expected Results *
Kidney Allograft Outcomes Registry—NCT03326076 (Prospective Observational)	CareDx sponsored Multicenter	4000 participants (18 years and older); planned surveillance biopsy vs unplanned surveillance biopsy	Multi-organ and bone marrow transplant recipient, identical twin organ recipient, pregnant, less than 14 days post-transplant	Biopsy-proven incidence of interstitial fibrosis/tubular atrophy, total number of biopsies—surveillance and diagnostic	Biopsy-proven transplant glomerulopathy, patient and graft survival, serum creatinine, eGFR, sensitivity and specificity of Allosure, NPV and PPV of Allosure, validation of KidneyCare	12/2025
Trifecta-Kidney cfDNA-MMDx Study—NCT04239703 (Prospective Observational)	University of Alberta—Natera, Inc, One Lambda	300 participants (all ages)	Multi-organ recipients	ddcfDNA measurements for TCMR, ABMR, AKI, CKI	DSA measurements, Renal Biopsy Results	12/2024; currently recruiting
Blood Biomarkers in Pediatric Kidney Transplant Recipients (Omnigraf)—NCT05477082 (Prospective Observational)	University of Minnesota	30 participants (up to 21 years)	-	Incidence of biopsy-proven ABMR; serum Creatinine	-	12/2023
Study for the Prediction of Active Rejection in Organs Using Donor-derived Cell-free DNA Detection (SPARO)—NCT03984747 (Prospective Observational)	Natera, Inc; Multicenter	500 participants (2 years and older); adult vs pediatric vs pregnant population	Identical twin organ recipient	Incidence of allograft rejection based on biopsy	cfDNA measurements	10/2028
Dd-cdDNA and Treg in Prediction of Kidney Transplant Acute Rejection—NCT05084768 (Prospective Observational)	Loma Linda University	150 participants (18 years and older); Rejection vs No Rejection groups	Multi-organ transplant recipient, +HIV, +HCV	Incidence of biopsy-proven ABMR	Incidence of graft failure	10/2026
Noninvasive Blood Test to Diagnose Acute Rejection After Kidney Transplantation (DART)—NCT02424227 (Prospective Observational)	CareDx sponsored Multicenter	401 participants (18 years and older)	Pregnant, multi-organ transplants, identical twin organ recipient	Incidence of ABMR, TCMR—clinical and subclinical	Serum GFR, allograft injury from BKV nephritis, CNI toxicity, Acute Pyelonephritis, Recurrent Disease	Completed 01/2019; no published results
Study for detection of donor-derived cell-free DNA after renal transplantation using Devysers NGS-based chimerism assay—NCT05226936 (Prospective Observational)	Sheba Medical Center	50 participants (20–70 years)	Multi-organ transplant recipient, graft loss within 3 months, enrolled in other study	Degree of chimerism of cd-DNA	-	03/2024; not yet recruiting
Donor-derived cell-free DNA for early diagnosis of antibody-mediated rejection—NCT04897438 (Randomized Interventional)	Charite University, Berlin, Germany	40 participants (18 or older)	Pregnant, coagulopathy, multi-organ transplantation, previous history of biopsy-proven ABMR, enrolled in another study	Time from DSA to biopsy-proven rejection, time from start of study to rejection	Sensitivity, specificity, ROC analysis of ddcfDNA for ABMR detection; GFR, albuminuria, mortality, severe infection, graft failure at 12 and 24 months, morbidity from biopsy, rate of ABMR, DSA at 0,12,24 months, immunosuppressive regimen	09/2024

Data retrieved from www.clinicaltrials.gov, accessed on 30 August 2022. * Due to optional uploading of published results to www.clinicaltrials.gov, some sources of published results may be missing from this table.

## Data Availability

Not applicable.
